# Phylogeography of Cold Water Soft Coral *Alcyonium* spp. (Anthozoa, Octocorallia: Alcyonacea) Between South America and the West Antarctic Peninsula

**DOI:** 10.1002/ece3.70522

**Published:** 2024-12-02

**Authors:** Paulina Bruning, Phillippe Archaumbault, Ignacio Garrido, Ander M. de Lecea, Simon A. Morley, Antonio Brante, Paula Ortiz, Leyla Cárdenas

**Affiliations:** ^1^ Takuvik, Quebec Ocean, Department of Biology Université Laval Québec Quebec Canada; ^2^ Centro FONDAP de Investigación en Dinámica de Ecosistemas Marinos de Altas Latitudes (IDEAL) Punta Arenas Chile; ^3^ Laboratorio Costero de Recursos Acuáticos de Calfuco (ICML), Facultad de Ciencias Universidad Austral de Chile Valdivia Chile; ^4^ South Atlantic Environmental Research Institute Stanley Falkland Islands; ^5^ Department of Environmental Sciences College of Agriculture and Environmental Sciences University of South Africa Pretoria Gauteng South Africa; ^6^ British Antarctic Survey Natural Environment Research Council Cambridge UK; ^7^ Departamento de Ecología, Facultad de Ciencias Universidad Católica de la Santísima Concepción Concepción Chile; ^8^ Centro de Investigación en Ecosistemas de la Patagonia (CIEP) Coyhaique Chile; ^9^ Instituto de Ciencias Ambientales y Evolutivas, Facultad de Ciencias Universidad Austral de Chile Valdivia Chile

**Keywords:** biodiversity, biogeography, divergence time, evolution, octocorals, phylogeny, Southern Ocean

## Abstract

The Antarctic marine environment has a unique geologic and climatic history that has contributed to the evolution of high species diversity. Given the current trend of environmental warming, understanding the history of Antarctic species is crucial for predicting the impact of climate change on ecosystem function. Soft corals are a group of striking presence in the benthic marine assemblages in the Southern Ocean, which is recognized as a biodiversity hotspot. DNA sequences (Cox1, mtMutS, and 28S rDNA) were utilized for molecular phylogenetic reconstructions, species delimitations, and divergence estimations to investigate the spatial patterns of genetic diversity in *Alcyonium* species in the southern South American‐Antarctic region. Significant genetic divergence was observed between regions, with a clear genetic break between South America and the West Antarctic Peninsula and the identification of four putative species. Divergence time estimates indicated that *Alcyonium*'s diversification began about 41.1 million years ago (Ma), coinciding with the opening of the Drake Passage and the formation of the Antarctic Circumpolar Current (ACC, ~42 Ma). This indicates that *Alcyonium* has persisted in situ for an extensive period, enduring a wide range of environmental conditions.

## Graphical abstract

Our study on genetic diversity in Alcyonium species across the southern South American‐Antarctic region revealed significant genetic divergence, indicating long‐term persistence and adaptation to varied environmental conditions since their diversification approximately 41.1 million years ago, coinciding with the opening of the Drake Passage and the formation of the Antarctic Circumpolar Current.

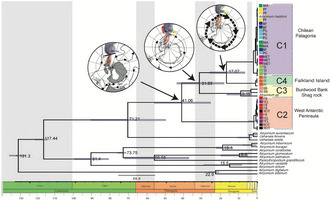

## 1 Introduction

Understanding the dynamics of marine ecosystems relies on comprehensive knowledge of species diversity and distribution patterns. Coral reefs are one of the most productive, diverse, and ancient ecosystems on Earth (Connell 1978; Quattrini et al. 2020); however, they face significant threats from climate change, with predictions of potential extinction within the next century (Carpenter et al. 2008; Holstein et al. 2022; Hughes et al. 2014). Consequently, it is crucial to establish baseline data on abundance, distribution, and diversity, especially considering the declines in population and species richness they are experiencing (Sebens 1994).

One group of corals, the Subclass Octocorallia, is the earliest diverged anthozoan group with an estimated divergence time of 544 million years ago (Jeon et al. 2019). Octocorallia encompasses over 3500 species of soft corals, sea fans, and sea pens (Williams and Cairns 2019). Octocorals are found in marine habitats worldwide (Cairns 2007) and play a critical role as foundation species, creating structurally complex three‐dimensional habitats that support numerous other invertebrate and fish taxa (Buhl‐Mortensen et al. 2010; Krieger and Wing 2002; Schweitzer and Stevens 2019). Like hard corals, they are susceptible to heat stress, bleaching, and mortality because of changing environmental conditions and habitat loss by climate change, sedimentation, and pollution (De'ath et al. 2012; Loya et al. 2001). However, compared to hard corals, knowledge about soft corals is extremely scarce, particularly in high‐latitude regions. Despite this, some recent studies have found new information about population abundance, distribution patterns, population ecology, species diversity, and evolutionary history (McCook et al. 2009; McFadden et al. 2011, 2006; Taylor and Rogers 2015).

Among the diverse class of Octocorallia, the order Alcyonacea stands out as one of the most diverse orders. Organisms within this order are sessile and form an integral part of benthic assemblages in many regions (Fabricius 1997). Soft corals belonging to the Alcyonacea group are dominant components of the benthic marine assemblage of the Southern Ocean (SO), with high abundance covering up to 75% of the substratum (Post et al. 2011; Slattery and Bockus 1997). These corals are primarily associated with biodiversity hotspots, particularly on vertical walls and crevices of hard substrata, in the seas surrounding the West Antarctic Peninsula (WAP, personal observation) and southern South America (SA) (Försterra, Häussermann, and Laudien 2015).

Within Alcyonaea, the genus *Alcyonium* includes small soft corals that form colonies of polyps connected by fleshy tissue. A wide range of growth forms are observed in Alcyoniidae (Lamouroux, 1812), from encrusting to lobate and digitate forms, as well as a plethora of sclerite shapes and arrangements (Alderslade 2000; Williams 2008). Sclerites provide shape and support, often characterized by their spiky appearance (Núñez‐Pons et al. 2013). Several species are known to be rich in bioactive compounds that provide defense against predators, are used during competition for space, and provide antifouling (Abdel‐Lateff et al. 2019; Núñez‐Pons et al. 2013). *Alcyonium* displays a diverse range of reproductive strategies, including gonochorism, hermaphroditism, and parthenogenesis, and utilizes both broadcast spawning and internal or external brooding of larvae (McFadden et al. 2001).

In recent years, the region encompassing Antarctica and its surroundings, including the southern South American continent, has garnered significant attention. Soft corals found in this region are specifically adapted to cold and dark environments. Furthermore, this region appears to be a potential center of endemism, with an increasing number of species being discovered, thereby enhancing its overall biodiversity and richness (e.g., Camps‐Castella et al. 2023; Goffredo and Dubinsky 2016; Van Ofwegen, Häussermann, and Försterra 2007; Zapata‐Guardiola and López‐González 2010). In *Alcyonium* species, taxonomic progress has been primarily based on morphological characteristics. Currently, six species of the genera *Alcyonium* have been described. One of these species, *Alcyonium antarcticum* (Wright 1889), is a lobate octocoral widely distributed in Antarctic and sub‐Antarctic regions, occurring at depths ranging from 25 m to nearly 642 m (Casas, Ramil, and Van Ofwegen 1997). *A. antarcticum* is locally abundant in protected habitats, often covered by a canopy of algae from the order Desmarestiales, as observed in the South Shetland Islands, but also on bare rock further south where no large canopy‐forming macroalgae are present (personal observation). In Pacific Patagonia, other *Alcyonium* species have been identified. *Alcyonium haddoni* (Wright 1889) has a geographical range spanning from 43° S to 51° S, *Alcyonium jorgei* (Van Ofwegen, Häussermann, and Försterra 2007) has been recorded in the continental fjords of the Northern Patagonian Zone and possibly once in the Central Patagonian Zone between 41° S and 48° S; *Alcyonium roseum* (Tixier‐Durivault, 1954) inhabits the channels of the Central Patagonian Zone, between 48° S and 51° S; and *Alcyonium glaciophilum* (Van Ofwegen, Häussermann, and Försterra 2007) occurs around 48° S. Additionally, *Alcyonium yepayek* (Van Ofwegen, Häussermann, and Försterra 2007) has been documented in channels of the Central Patagonian Zone, between 48° S and 50° S. These lobate colonies are typically found on rocky substrates at very shallow depths (< 30 m), with the exception of *A. haddoni*, which exhibits a wider depth range of 6–350 m (Van Ofwegen, Häussermann, and Försterra 2007). In Atlantic Patagonia, *Alcyonium* corals are reported to be one of the most abundant soft coral groups. However, their taxonomy is currently only classified at the genus level, and thus their presence is commonly documented as *Alcyonium* sp. in the existing literature (Schejter and Bremec 2019; Schejter et al. 2016).

Despite their ecological importance and regional abundance. there is limited information regarding the geographical distribution (Cárdenas et al. 2008; Casas, Ramil, and Van Ofwegen 1997; Van Ofwegen, Häussermann, and Försterra 2007) and a lack of genetic information about *Alcyonium* available for the Patagonia‐Antarctic region, with only two known DNA sequences. One sequence is attributed to *A. haddoni* from Chilean Patagonia, featured in a phylogeny by McFadden et al. (2006). The second sequence corresponds to *Alcyonium* sp., sampled near Shag Rocks in the Scotia Arc of Antarctica as part of a comprehensive study on the chemistry and bioactivity of these corals (Limon et al. 2022). Molecular tools, such as DNA information combined with spatial sampling, provide an opportunity to investigate the actual biodiversity and historical diversification of this group (Quek and Huang 2022). In the Alcyonacea order, distinguishing species limits and morphological plasticity can sometimes only be achieved using molecular techniques (Baco and Cairns 2012; McFadden and Van Ofwegen 2013). Previous studies on the evolutionary history of the Antarctic fauna have revealed strong affinities between Antarctica and southern South American regions, particularly with the tip of South America, also known as the “Antarctic‐South American Connection” (e.g., Allcock and Strugnell 2012; González‐Wevar et al. 2017; Thatje 2012). This connection is explained by the historical continental separation of Antarctica and South America, the opening of the Drake Passage, and subsequent changes in oceanographic circulation (Crame 1999). Three hypotheses have been postulated to explain the present species distribution. The vicariant hypothesis relates biodiversity patterns to the continental separation of the Antarctic, followed by the opening of Drake passage 42 million years ago (Ma) (Clarke and Crame 2010; Crame 1999; Halanych and Mahon 2018) and the subsequent deepening of the Tasman Sea (34–33 Ma) (Stickley et al. 2004). These changes modified the oceanographic circulation of the Southern Ocean, and, around the Oligocene/Eocene boundary, the Antarctic Circumpolar Current (ACC) and the Antarctic Polar Front (APF) formed (32 Ma). The dispersal hypothesis highlights the function of the ACC in transporting marine organisms around the Antarctic, especially in taxa with high dispersal ability (Avila et al. 2020; González Wevar et al. 2018; Macaya et al. 2020). A third hypothesis, which also explains the eurybathy of many species inhabiting the Southern Ocean, is the connectivity hypothesis that invokes the Scotia Arc as providing intermediate dispersal points or ‘stepping stones’ across the Drake Passage (Clarke et al. 1992). Up to now, few genetic studies on soft corals in the Southern Ocean have been published, and they have been mainly focused on deep octocorals of the Family Primnoidae (Dueñas et al. 2016; López‐González 2020; Taylor and Rogers 2015). A study including 64 species representing 25 genera of the common deep‐sea octocoral family Primnoidae suggests a Pacific origin, indicating that Primnoidae sub‐Antarctic diversity is the result of secondary species radiation. There is also evidence for a subsequent range extension of sub‐Antarctic lineages into deep water areas of the Indian and Pacific Oceans. The diversification of Primnoidae (mean 52 Ma) predates the initiation of the ACC in support of the vicariant hypothesis, meaning Primnoidae have been in situ for an extensive period of time and over a large range of environmental conditions (Taylor and Rogers 2015). Given the lack of genetic information on the soft coral genus *Alcyonium* from the Patagonia‐Antarctic region, this study aims to fill this knowledge gap. We then aim to enhance our understanding of the evolutionary history of this group in cold waters. Based on the current evidence, we expect that the diversification process in *Alcyonium* may share similarities with that observed in Primnoidae, leading us to anticipate that its evolutionary history will best fit with the vicariant hypothesis. Using a phylogeographic approach, we aim to provide new insights about: (i) diversity patterns in populations of *Alcyonium* along the southern South America (SA) and the West Antarctic Peninsula (WAP); (ii) genetic delimitation of species currently present in SA and WAP; and (iii) diversification history of this widespread component of the biodiversity in a region that has been relatively understudied despite its global importance. Additionally, our findings will have implications for conservation and management efforts related to this ecologically important group in this unique marine environment.

## 2 Methods

### 2.1 Sampling and Molecular Data Collection

**FIGURE 1:**
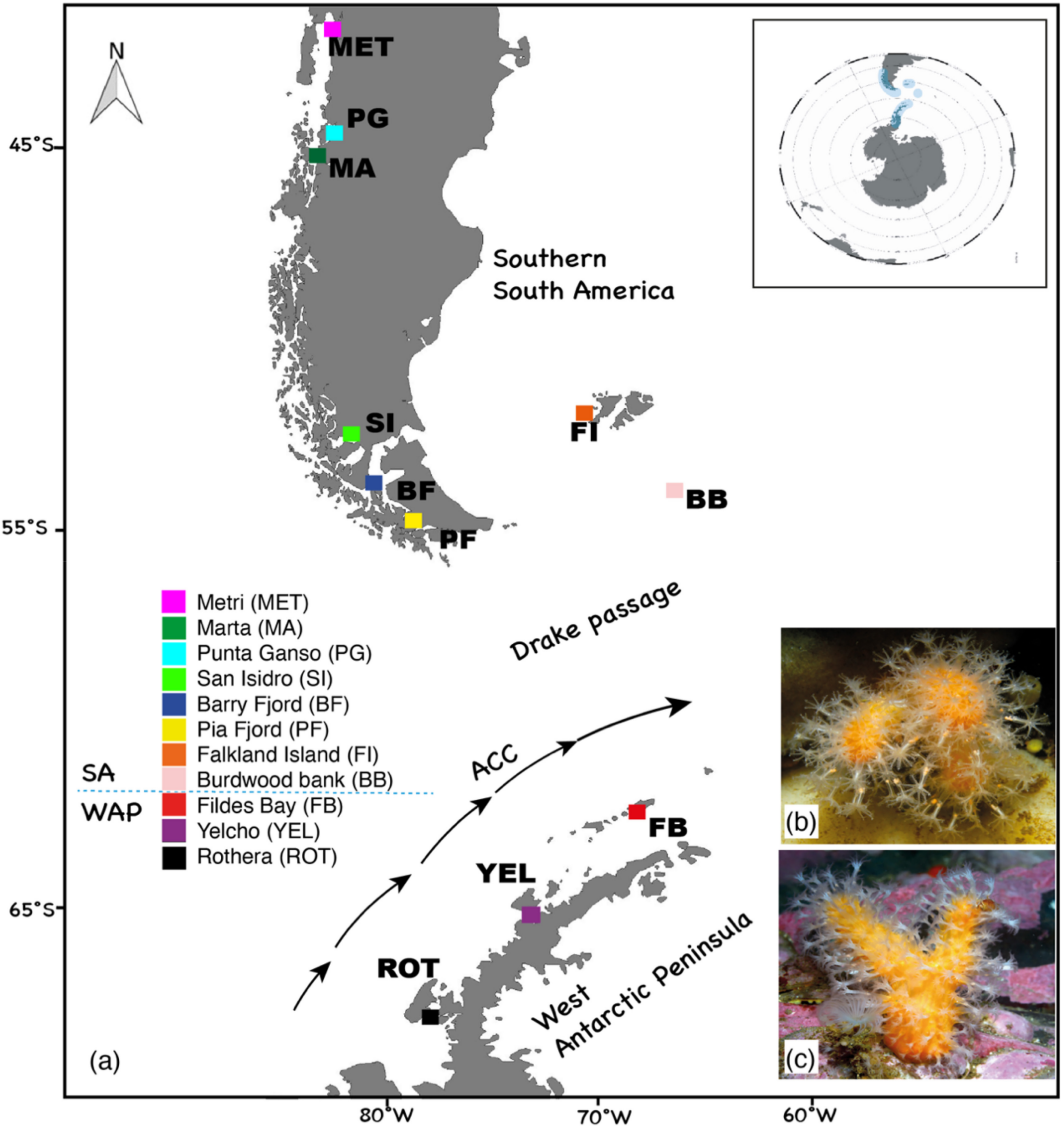
(a) Study area and sampling localities for the genus *Alcyonium* between South America (SA) and the West Antarctic Peninsula (WAP). A schematic representation of the Antarctic Circumpolar Current (ACC) is included for reference, illustrating the simplified flow pattern in the region. (b) A specimen of *Alcyonium antarcticum* collected from Fildes Bay, King George Island. (c) Specimen of *Alcyonium haddoni* collected from San Isidro, Strait of Magellan.

Cold soft corals of *Alcyonium* spp. were collected between 2018 and 2020 at different localities in the Southern Ocean (Figure 1), with samples from the South American (SA) region including the Chilean Patagonia (PAT) with Metri (MET), Punta Ganso (PG), Marta (MA), San Isidro (SI), Barry Fjord (BF), Pia Fjord (PF), the Falkland Islands (FI), and Bordwood Bank (BB) and samples from the Western Antarctic Peninsula (WAP) region including Fildes Bay (FB), Yelcho (YEL), and Rothera (ROT) (Figure 1; Table 1). All except one collection were made by SCUBA diving, at depths ranging from 5 m to 40 m on the rocky reef. The BB locality sampling was carried out at 395 m depth using a mini Agassiz Trawl (AGT) with a mouth width of 1.25 m and a mesh size of 1 cm. Immediately after collection, specimens were photographed in the laboratory and then preserved in 70% ethanol with subsamples for DNA analysis fixed in 90% ethanol and stored at room temperature until further examination. A total of 60 samples from 11 localities were used for DNA sequence analyses (Table 1).

**TABLE 1:** Sampling sites information for *Alcyonium* spp. collected for this study.

Region	Site	Code	Coordinates	*N*	Depth	Cox1+mtMuts		28S rDNA	
						*h*	π	*h*	π
SA	Metri, Northern Patagonia	MET	41°40′ S/72°39′ W	5	40	1	0.0000	1	0.0000
	Punta Ganso, Aysen	PG	44°44′ S/72°44′ W	5	10–25	1	0.0000	1	0.0000
	Marta, Aysen	MA	44°50′ S/72°0.8′ W	5	10–25	1	0.0000	1	0.0000
	San Isidro, Magellan Strait	SI	53°47′ S/70°58′ W	9	20–40	1	0.0000	1	0.0000
	Barry Fjord, Beagle Channel	BF	54°36′ S/69°18′ W	4	20–40	1	0.0000	1	0.0000
	Pia Fjord, Beagle Channel	PF	54°56′ S/67°7′ W	3	20–40	1	0.0000	1	0.0000
	Falkland Island, Bird Island	FI	52°10′ S/60°55′ W	6	15–20	1	0.0000	1	0.0000
	Burdwood Bank, oceanic point	BB	54°58′ S/57°45′ W	2	395	2	0.0024	1	0.0000
				39	Total	7	0.0029	3	0.0052
WAP	Fildes Bay, King George Island	FB	62°12′ S/58°49′ W	10	5–20	1	0.0000	1	0.0000
	Yelcho, Doumer Island	YEL	64°47′ S/63°28′ W	3	5–20	1	0.0000	1	0.0000
	Rothera, Adelaide Island	ROT	67°35′ S/68°12′ W	8	5–20	3	0.0006	2	0.0014
				21	Total	4	0.0004	2	0.0022

*Note:* The sampling sites are categorized into two regions: South America (SA) and the West Antarctic Peninsula (WAP). Each site is identified by an abbreviation (code), geographic coordinates, the numbers of individual sequenced (N), depth, and genetic diversity indices for both mitochondrial mtMutS+Cox1 (mt) and 28S genes, including the number of haplotypes (*h*) and nucleotide diversity (π).

Mito‐nuclear sequence analyses. DNA extraction was performed from 20 mg to 30 mg of tissue using the EZNA Kit (EZNA, Inc.) according to the manufacturer's protocol. The quality of the DNA extracts was assessed visually on 1.0% agarose gels, and the DNA concentration (ng/μL) was measured on a Nanodrop 1000. Three gene regions were targeted: two mitochondrial genes Cox1 (McFadden et al. 2004), mtMutS (France and Hoover 2002; Sánchez et al. 2003), and one nuclear gene 28S rDNA (adapted from McFadden and Van Ofwegen (2012)). PCRs were performed using a thermocycler (Multigene Optimal, Labnet) following the protocol described by Taylor and Rogers (2015). Amplicons were sequenced by the Sanger method using an ABI PRISM 3100 Genetic Analyzer at the core facility of Universidad Austral de Chile (www.australomics.cl). Sequences were assembled into contigs, edited, and aligned using the program Generous 6.0.5. Alignments and base composition of nucleotide sequences were analyzed for each marker independently in MEGA 7.0 using MUSCLE with standard settings. Soft coral sequences were deposited in the public database GenBank. Samples used in the analyses are detailed in Table 1, and the sequence accession numbers are listed in Table S1.

Genealogical relationships were visualized through a haplotype network based on a mitochondrial gene (Cox1+mtMutS) and one form of nuclear gene (28S rDNA). For *Alcyonium* populations, a haplotype network was estimated using maximum parsimony networks in MEGA and visualized in NETWORK version 10.2 (Fluxus Technology, Ltd).

The concatenated (Cox1+mtMutS) and 28S rDNA alignments were used to infer two phylogenies. Based on previous works (Brockman and McFadden 2012; Lau and Reimer 2019; McFadden et al. 2011; Parrin et al. 2012), we selected a set of species of the same genus and family (*Gersemia rubiformis*, *Gersemia juliepacardae, Gersemia antartica, Alcyonium digitatum*, *Alcyonium siderium, Alcyonium variabile, Alcyonium dolium, Alcyonium palmatum, Alcyonium glomeratum, Alcyonium hibernicum, Alcyonium corralloides, Alcyonium bocagei, Alcyonium haddoni, Parerythropodium grandiflorum, Alcyonium aurantiacum, Ushanaia fervens, Ushanaia solida*) whose sequences were available in Genbank. In addition, the species *Azoriella bayeri* was selected as an outgroup (Kessel et al. 2022). For more details, see Table S1.

Phylogenetic tree reconstructions were performed using Maximum Likelihood (ML) and Bayesian Inference (BI) in the software MEGA 7, IQ‐TREE (Nguyen et al. 2015) at the IQ‐TREE web server and MrBayes v.3.2 available in the CIPRES Science Gateway, respectively. The best‐fit model of nucleotide substitution was evaluated in jModeltest 2.1.3. The selected model, GTR+I+G, was set in genealogical reconstruction for ML and BI analysis. Nodal support for ML analyses was inferred using nonparametric bootstrapping (BS) with 1000 pseudo‐replicates (Felsenstein 1981). Bayesian‐inference posterior probabilities (PP) were estimated using the Metropolis‐coupled Markov‐chain Monte‐Carlo algorithm (MCMC) running four chains for 50 × 10^6^ generations, with trees sampled every 1000 generations. The initial 10% of the values were discarded (burn‐in), and posterior probabilities were estimated as the fraction of trees showing a particular clade. Finally, posterior probability density was summarized as a maximum clade credibility tree using TreeAnnotator v.1.6.1 (http://beast.bio.ed.ac.uk/TreeAnnotator) and visualized using FigTree v.1.4.3 (http://tree.bio.ed.ac.uk/software/figtree). Similarly, two databases were used for species delimitation, one composed of mitochondrial genes (Cox1+ mtMutS) and one based on nuclear gene 28S rDNA, analyzed separately. This analysis was performed using three independent methods: Automatic Barcode Gap Discovery (ABGD) (Puillandre et al. 2012), Poisson Tree Processes (PTP) (Zhang et al. 2013), and General Mixed Yule Coalescence (GMYC) (Pons et al. 2006). The ABGD method relies on genetic distances to sort DNA sequences into primary species hypotheses (PSHs) using an a priori defined threshold (i.e., the “barcode gap”). The Bayesian PTP infers putative species boundaries starting from a phylogenetic tree and counting the number of substitutions inferred by the length of the branches. Finally, the GMYC method is based on the likelihood that delimits species‐adjusting models ramified intra‐and inter‐species to a reconstructed gene tree (Reid and Carstens 2012). Although the three methods use different input information to delimit species (i.e., sequence alignments or phylogenetic trees), they all provide information on the number of different candidates/hypothetical species in a collection of sequences. For the ABGD approach, we ran the analysis online (http://wwwabi.snv.jussieu.fr/public/abgd/abgdweb.html) with default options using the Kimura 2‐parameters as an evolutionary model. Bayesian PTP analysis was performed in the bPTP web server (http://species.h‐its.org/ptp/) using the rooted “best tree” generated by RAxML. The analysis was run for 500,000 generations using a random number sequence and a thinning of 100. A quarter of the sampled trees were discarded as burnt‐in.

Species delimitation probability values were calculated with the Bayesian method by considering the frequency of the nodes across the sampling. For GMYC, single threshold model was carried out using the “Species Limits by Threshold Statistics” approach (SPLITS v1.0‐19) in the R program (www.r‐project.org). Also, a Bayesian implementation of the GMYC model, bGMYC, was used in the R package (Reid and Carstens 2012). This Bayesian analysis sampling over the posterior distribution of gene trees allows uncertainty in topology and branch lengths to be reflected in posterior parameter estimates. A range of probabilities > 0.95 was considered solid evidence that the groups compared were conspecific, while a range of probabilities < 0.05 strongly suggested that the groups compared were not conspecific.

To perform divergence time analysis, the mitochondrial gene mtMutS was used because this mitochondrial gene shows a relatively faster evolutionary rate among the mitochondrial genome of octocorals (France 2007; Herrera, Baco, and Sánchez 2010), which have evolutionary rates 10–100 times slower than those of other metazoans (Shearer et al. 2002). A relaxed molecular clock analysis was implemented for mtMutS sequences using an uncorrelated‐lognormal (ucln) model of molecular evolutionary rate heterogeneity and the GTR+I+G substitution model implemented in BEAST 2.3.1 (Bouckaert et al. 2014). A Yule model prior was used for branching rates in the phylogeny. The analysis was run with four chains, twice each for 50 × 10^6^ generations, and trees were sampled every 1000 generations. Given the absence of suitable *Alcyonium* fossils to calibrate our analysis, we employed two ways to establish a dated phylogeny. Firstly, we utilized a mutation rate of 0.25% per million years, consistent with prior studies in species of the genus *Paramuricea* (Poliseno et al. 2017). Additionally, we incorporated established divergence times for specific *Alcyonium* species (Simone 2013), calibrated with available Corallidae fossils dating back to 83.5 Ma. The convergence of model parameters was estimated by plotting the marginal posterior probabilities versus the generations in Tracer v.1.5 (http://beast.bio.ed.ac.uk/Tracer), and effective sample‐size values were calculated for each parameter to ensure adequate mixing of the MCMC (ESSs > 1000). Subsequently, results were summarized to produce a maximum credibility tree using TreeAnnotator v.2.4.8. The first 1000 trees (20%) of each run were discarded as burn‐in. A geological timescale tree was plotted using the packages strap (Bell and Lloyd 2015) and phytools (Revell 2012) in the R statistical package v. 3.1.2 (R Core Ream 2008).

## 3 Results

### 3.1 Mito‐Nuclear Sequences Analyses

A total of 60 individuals from 11 localities were analyzed (Table 1). The mitochondrial gene Cox1 had a total length of 756 base pairs (bp), the mitochondrial gene mtMutS had a total length of 735 bp, and the nuclear gene 28S rDNA had a total length of 830 bp.

**FIGURE 2:**
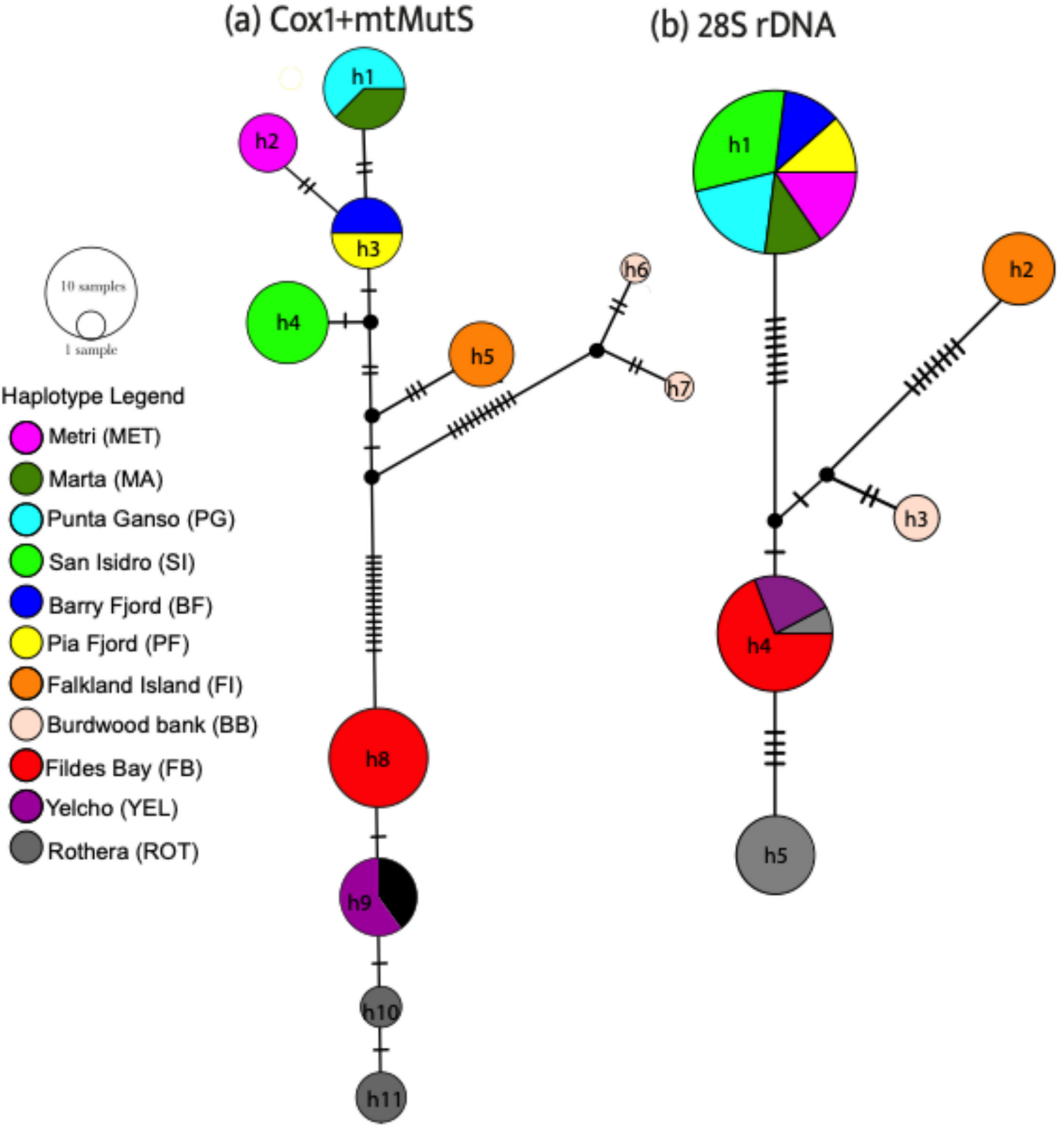
Haplotype network, with haplotypes color‐coded to match their corresponding geographical distribution (a) inferred from the mtDNA (Cox1+mtMutS) and (b) inferred from 28S rDNA.

The genealogical reconstruction of the haplotype network for the mitochondrial genes (Cox1 and mtMutS) revealed 11 different haplotypes (Figure 2a). Seven were present in SA; h1 was shared by Punta Ganso (PG) and Marta (MA), and h3 by Barry (BF) and Pia Fiord (PF). Others SA localities showed only private haplotypes: h2 for Metri (MET) and h4 in San Isidro (SI). These localities from Chilean Patagonia (PAT) are separated from the private haplotype (h5) Falkland Island (FI) by five mutational steps and are 15 mutational steps from two haplotypes (h6 and h7) from Burdwood Bank (BB). Meanwhile, four haplotypes were found in the WAP with h8 as a private haplotype from Fildes Bay (FB) and h10 and h11 from Rothera (ROT). The only exception was h9, which was shared by Yelcho (YEL) and ROT. This analysis revealed two main groups for mitochondrial genes separated by 18 mutational steps, with a 1.21% difference in nucleotide sequence indicating a genetic divergence between SA and the WAP (Figure 2a).

For 28S rDNA, five haplotypes were identified. Among these, h1 was a shared haplotype present in MET, MA, PG, SI, BF, and PF. The h1 was separated by 17 mutational steps (2.05% difference) from the private haplotype of FI (haplotype h2) and by 11 mutational steps (1.33% difference) from BB (haplotype h3). Additionally, two haplotypes were found in the WAP, with h4 exhibiting the highest frequency among FB, ROT, and YEL and separated by four mutational steps from h5, a private haplotype from ROT (Figure 2b).

**FIGURE 3:**
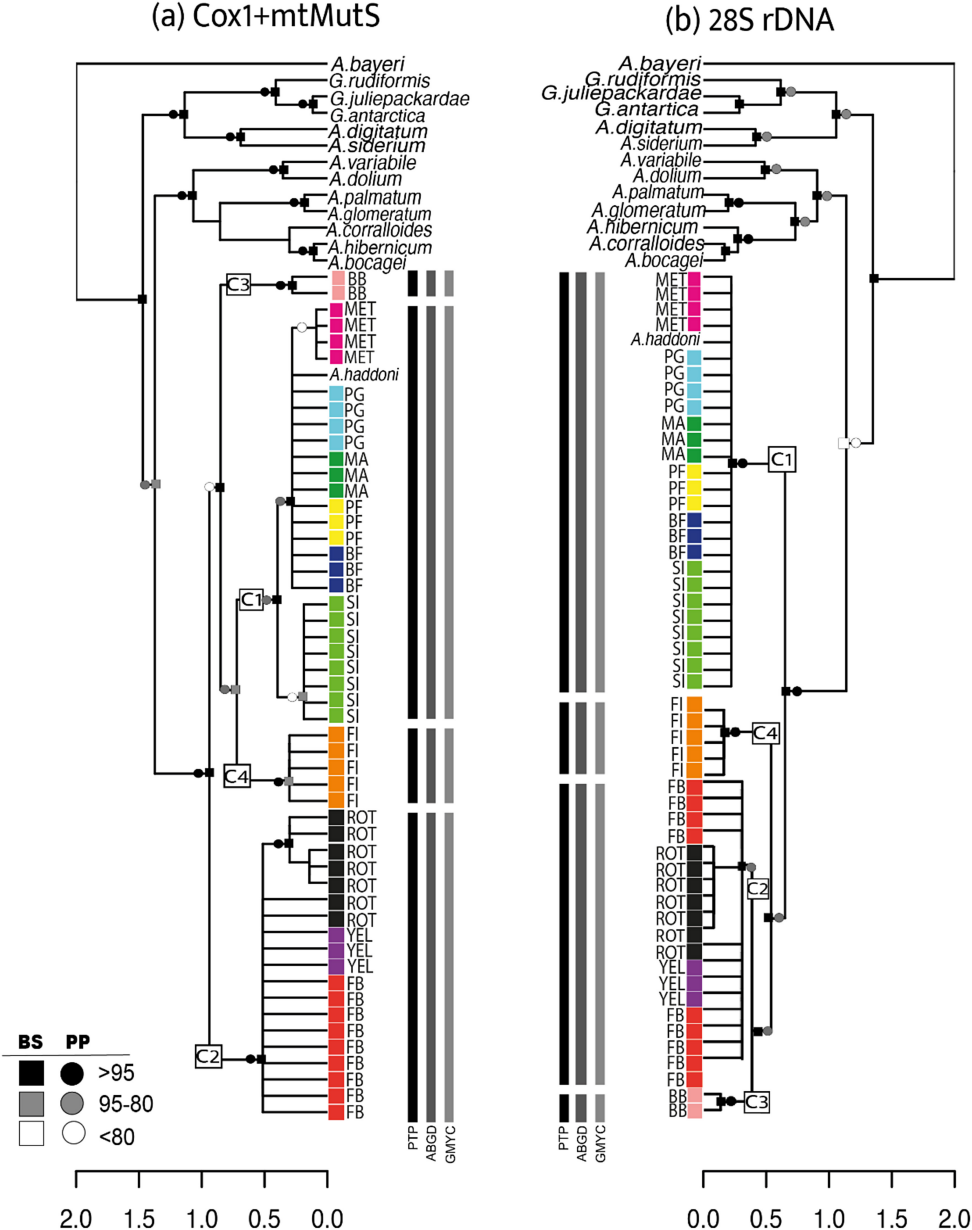
Phylogenetic tree and species delimitation analyses for *Alcyonium* spp. inferred from (a) the mtDNA (Cox1 + mtMutS) and (b) 28S rDNA. Maximum likelihood bootstrap support (BS) and Bayesian posterior probability (PP) values are represented by shaded squares and circles along the nodes. The tree was rooted with *Azoriella bayeri*. Major clades are annotated as squares on branches: C1 for Chilean Patagonia, C2 for West Antarctic Peninsula, C3 for Burdwood Bank, and C4 for Falkland Islands. The results of species delimitation analyses are depicted by black bars.

Two phylogenetic reconstructions based on mitochondrial (Cox1+mtMutS) and nuclear (28S rDNA) sequences identified a monophyletic group for the *Alcyonium* specimens in our study, supported by 100% BS and 1.0 of PP. The phylogenetic tree based on mitochondrial genes (Figure 3a) recovered clade 1 (C1) including specimens collected across PAT, a sequence from Genbank of the species *A. haddoni*, and samples from MET, BF, PF, SI, PG, and MA. A second clade (C2) grouped individuals exclusively from the WAP with samples from ROT, YEL, and FB. A third clade (C3) included those individuals collected from BB, and the fourth clade (C4) included individuals from FI. The phylogenetic tree based on the nuclear gene recovered four main clusters with similar general distribution (Figure 3b): i.e., C1 included samples from PAT, C2 included samples from WAP, C3 was composed by samples from BB, and C4 incorporated individuals collected in FI. However, different topologies were recovered. In the mitochondrial tree, C2 diverges from the other three clades, while in the nuclear phylogeny, it is C1 that diverges. In addition, the summary bars of species delimitation (bars in Figure 3) showed that, independently of the species‐delimitation method, the analysis recovered four groups concordant with the cluster recovered by phylogenetic analysis.

**FIGURE 4:**
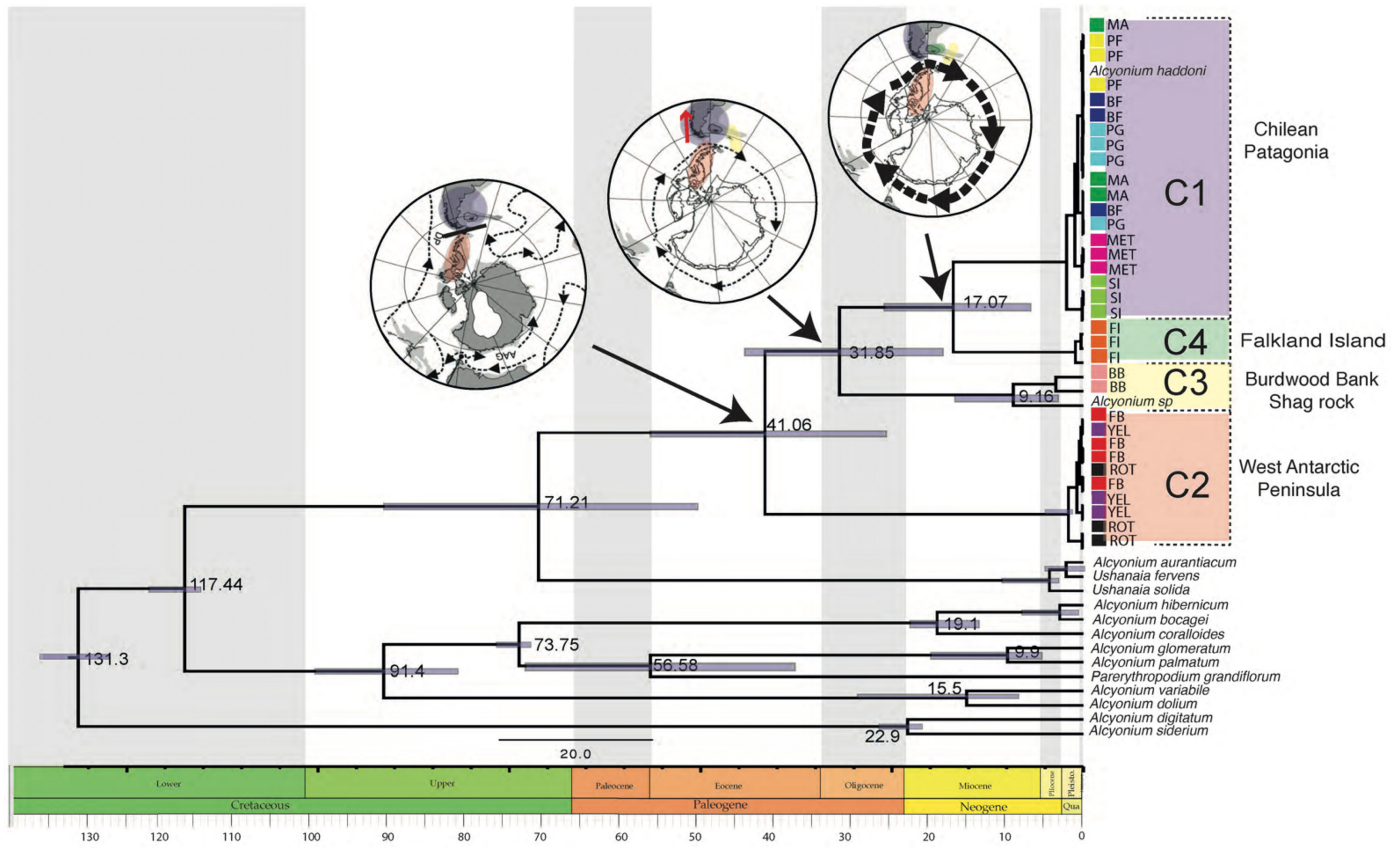
Chronogram of the maximum clade credibility tree for the mtMuts dataset, estimated using a conservative mutation rate of 0.25% per million years. Bars at the nodes represent the 95% highest posterior density intervals, highlighting the uncertainty around estimated divergence times. The scale bar indicates time in millions of years. The accompanying maps illustrate the biogeographic implications of tectonic and oceanographic changes over the past 50 million years. Modified from Cantrill and Poole (2012).

Divergence time estimation (Figure 4) using the most variable gene (mtMutS) suggests that the diversification process began at around 41.1 (range 56–25) Ma during the late Eocene to Oligocene, with the initial separation occurring between the WAP and SA clades. Following this, in SA, the BB clade diverged from the rest of the SA samples (FI and PAT) at 31.8 (43–18) Ma. The most recent diversification event is estimated to have occurred between the FI and PAT around 17 (25–6) Ma.

## 4 Discussion

To date, studies investigating the phylogeny and reproductive biology of the genus *Alcyonium* have mainly focused on the Northern Hemisphere, especially in Europe and North America (Erickson et al. 2021; McFadden et al. 2001; McFadden and Van Ofwegen 2013). In the present work, we report for the first time the spatial genetic pattern of the genus *Alcyonium* in the southern South America‐Antarctic region. Our analyses combining mitochondrial and nuclear DNA markers revealed genetic differentiation between samples from SA and the WAP and identified four genetic lineages strongly suggesting different species of *Alcyonium*.

Interestingly, our findings also demonstrate that samples originating from the Southern Hemisphere form a separate clade from those in the Northern Hemisphere. *Alcyonium* species from the Northern Hemisphere exhibit a closer genetic relationship with the genus *Gersemia* than with congeneric *Alcyonium* from the Southern Hemisphere. This reinforces the notion that samples from the Southern and Northern Hemispheres have distinct evolutionary trajectories. Previous studies have already indicated that *Alcyonium* clearly constitutes a paraphyletic group that does not include all the descendants of a common ancestor (Kessel et al. 2022). From an evolutionary point of view, this is an important finding that could change our understanding of the history and the diversification of this group. At present, more than 60 nominal species of *Alcyonium* remain unrepresented in molecular phylogenies (Kessel et al. 2022). In our study, we have included at least four new lineages from a previously unrepresented Southern Hemisphere area. Our findings suggest the existence of evolutionary independent units (EIUs) within the genus *Alcyonium*, each specific to particular geographic regions. These EIUs require comprehensive investigation using morphological, biogeographical, and genetic data.

Reconstructed relationships among groups were incongruent between mitochondrial and nuclear phylogenetic reconstructions. The most simple explanation for this discordance could be because the nuclear markers used here (28S rDNA) may not be variable enough to reflect the recent divergence (Toews and Brelsford 2012). On the other hand, incomplete lineage sorting, selection, hybridization, and introgression could be alternative explanations for this pattern (Funk and Omland 2003; Quattrini et al. 2023). In the case of anthozoans, introgressive hybridization has been suggested as a significant mechanism for generating species diversity (Hogan et al. 2023; Quattrini et al. 2023). To clarify the biogeography pattern associated with the spatial genetic diversification in these four *Alcyonium* lineages will require new molecular diagnostic tools, a wide genomic screening perspective, and a more exhaustive sampling design.

In our study, the species delimitation analysis supports the occurrence of four EIUs, and based on our data, we will refer to these as putative species. We followed the approach proposed by Korfhage et al. (2022), who tested the efficacy of genetic markers in delimiting coral species, which proved to be the most reliable and suitable method for discriminating coral morphospecies (Korfhage et al. 2022). In the WAP, a unique clade was detected where the only described and accepted species is *Alcyonium antarcticum*. This species has been described as widely distributed in the Antarctic between 25 m and 642 m depth (Schories and Kohlberg 2016; Verseveldt and Van Ofwegen 1992), and we assume that the new genetic information from this study belongs to this species. Nevertheless, an integrative taxonomic approach would help to obtain a more robust species classification. In Chilean Patagonia, based on genetic similarity, we detected a clade that corresponds to the species *Alcyonium haddoni* (Genbank accession GU355974). In addition, the geographic distribution of samples analyzed here coincides with those described for this species, which is the most abundant and common *Alcyonium* in this region bathymetrically, distributed from 5 to 315 m depth (Van Ofwegen, Häussermann, and Försterra 2007) and latitudinally from 43° S to 55° S in Chilean Patagonia (Cárdenas et al. 2008; Casas, Ramil, and Van Ofwegen 1997; Van Ofwegen, Häussermann, and Försterra 2007).

Two new putative species were also clearly identified, one in Burdwood Bank (BB) and another independent clade was present in the Falkland Islands (FI). The level of genetic differentiation is robust enough to be assigned as a putative species (Ence and Carstens 2011; Fujisawa and Barraclough 2013; Jones 2017; Zhang et al. 2013). Furthermore, the level of mitochondrial divergence between SA and WAP regions was (~1.21%), surpassing the proposed 1% threshold for mtMutS‐based species delimitation in octocorals (McFadden et al. 2011).

The Burdwood Bank (BB) is located in the Scotia Sea, and the putative species identified in this location have a genetic divergence of 1.82% with SA and 1.07% with the WAP. A sequence of *Alcyonium* sp. (accession OP429120) from Shag Rock locality in the Scotia Arc recovered from Genbank also aligns within the C3. It is interesting to note that the patterns of divergence reveal that BB specimens were more similar to Shag Rock than FI, PAT, or WAP specimens, despite the greater geographic distance separating BB and Shag Rock. The Scotia Sea islands seem to play an important role in the evolution of Southern Ocean biota (Linse et al. 2006), and BB is formally recognized as a deep sub‐Antarctic area of ecological importance and an oceanic hot spot of benthic biodiversity (Schejter et al. 2016). One of the most abundant biomes on the BB is mainly structured by cold‐water coral communities (Schejter and Bremec 2019). Moreover, previous coral studies in the Scotia Sea islands have reported high octocoral biodiversity. However, in these studies, specimens of the genus *Alcyonium* have only been described as *Alcyonium* sp. (Schejter et al. 2016). Additional analyses, including morphological examinations, may be necessary to confirm the taxonomic status of these two putative species. The taxonomic identification in octocorals is intricate, demanding specialized taxonomic expertise (Pérez et al. 2016); however, the genetic resources generated here will provide more detailed insights into these species, which could be crucial for accurate taxonomic classification.

Molecular analysis provides a valuable source of information for inferring the pattern and processes of diversification and to test the Antarctic‐southern South American connection. Our results suggest that these four putative species of *Alcyonium* were separated from the basal group around 41.06 Ma. The diversification began with the separation between SA and the WAP, followed by the separation of the BB clade around 31.85 Ma from the rest of SA, and with the most recent diversification event occurring between the FI and PAT around 17.07 Ma. *Alcyonium* includes different lineages that have been separated before the initiation of the ACC, supporting the vicariant hypothesis for the Antarctic‐southern South American connection. Finally, we suggest that the connectivity hypotheses should be further examined given that our results show an important role for the Scotia Arc in providing historical intermediate points in the diversification process of *Alcyonium* lineages (Clarke et al. 1992).

The observed diversification of *Alcyonium* in SA is probably driven by oceanographic changes associated with the period of intensification of the ACC circulation ~14 Ma (Lawver and Gahagan 2003) and the cooling of the Southern Ocean. This result is congruent with those reported for other soft coral communities from the ACC; for example, the diversification of the bottlebrush deep‐sea octocorals was estimated at around 6.6–20.3 Ma, also corresponding to the Miocene (Dueñas et al. 2016). The middle Miocene Climatic Transition (MMT) is considered an important period of oceanographic and climatic change in the SO, associated with the intensification of the Antarctic Circumpolar Current (ACC) and the re‐establishment of Antarctic continental ice sheets (Lawver and Gahagan 2003; Zachos et al. 2001). Furthermore, recent studies in the Scotia Sea indicate that a remnant volcanic arc, currently submerged, may have formed a barrier to deep ocean circulation eastward until the mid‐Miocene (about 11.2 Ma (Dalziel et al. 2013)), when the establishment of a deep ACC was achieved, ultimately separating the Antarctic and South American benthos (Poulin et al. 2014). These results help us to infer the role of geological and oceanographic processes in the area, where the persistence of a submerged volcanic arc along the central Scotia Sea after the mid‐Miocene (Dalziel et al. 2013) may result in a complex interplay between genetic isolation and divergence processes in SA, as was previously suggested by Poulin et al. (2014) with the connectivity hypothesis.

In summary, this study allowed us to advance our understanding of the evolutionary processes that have molded the extant soft coral biodiversity across the Southern Ocean. Results presented reveal four genetic lineages for *Alcyonium* and support the role of geoclimatic processes in the diversification of Alcyonacean species in both the West Antarctic Peninsula and South American regions. We support a complex interplay between environmental changes driven by climate and oceanography that has characterized the Southern Ocean throughout the Miocene, particularly since the Middle Miocene, and profoundly influenced the evolution and biogeography of the soft coral species studied here.

For future research, it is crucial to conduct a comprehensive taxonomic revision of the clades identified in this study in order to enhance our understanding of the marine biodiversity in the cold waters of the Southern Hemisphere. This will also potentially contribute to the ongoing global systematic reviews of Octocorallia. Additionally, there is a pressing need to expand our knowledge on this group of corals in the Southern Ocean, as limited information is available regarding their spatial distribution, ecology, reproduction, habitat associations, and vulnerability to anthropogenic threats and environmental changes.

## Author Contributions

**Paulina Bruning:** conceptualization (lead), data curation (supporting), formal analysis (lead), investigation (lead), methodology (lead), writing – original draft (lead), writing – review and editing (lead). **Phillippe Archaumbault:** conceptualization (supporting), funding acquisition (supporting), investigation (supporting), resources (supporting), supervision (supporting), writing – review and editing (supporting). **Ignacio Garrido:** investigation (supporting), methodology (supporting). **Simon A. Morley:** writing – review and editing (supporting). **Antonio Brante:** investigation (supporting), writing – review and editing (supporting). **Ander M. de Lecea:** methodology (supporting). **Paula Ortiz:** data curation (supporting). **Leyla Cárdenas:** funding acquisition (supporting), investigation (supporting), methodology (supporting), resources (equal), supervision (supporting), writing – review and editing (equal).

## Conflicts of Interest

The authors declare no conflicts of interest.

## Supporting information


**Table S1.** GenBank accession numbers for octocoral DNA sequences used in phylogenetic analysis.
**Table S2.** Pairwise genetic distance matrix based on mtDNA (Cox1+mtMutS; below the diagonal) and 28S rDNA (above the diagonal) gene sequences. Calculated using the MEGA3 software. The clades are as follows: 1. Clade 1—Chilean Patagonia; 2. Clade 2—West Antarctic Peninsula; 3. Clade 3—Burdwood Bank; and 4. Clade 4—Falkland Islands.

## Data Availability

The sequences of dataset were deposited in Genbank repository (https://www.ncbi.nlm.nih.gov/nucleotide/): Cox1(OP797670, OP797671, OP797672, OP797673, OP797674, PP101875, PP101876, OP797666, OP797667, OP797668, OP797669); mtMutS (PP182331, PP182332, PP182333, PP182334, PP182336, PP182337 PP182335 PP182330, PP182327, PP182328, PP182329), and 28S(OP799354, OP799355, OP799356, OP799357, OP799358, OP799353, OP799350, OP799351, OP799352, OR785765, OR785766). Additionally, the code used for data analysis is available in (Table S1).
