# Supplementary material for: Phylogeography of Cold Water Soft Coral Alcyonium spp. (Anthozoa, Octocorallia: Alcyonacea) Between South America and the West Antarctic Peninsula
Source: Ecol Evol. 2024 Dec 2;14(12):e70522. doi: 10.1002/ece3.70522 (PMC11612023; doi:10.1002/ece3.70522)
Supplement: Supplementary file 1 — Table S1. GenBank accession numbers for octocoral DNA sequences used in phylogenetic analysis. Table S2. Pairwise genetic distance matrix based on mtDNA (Cox1+mtMutS; below the diagonal) and 28S rDNA (above the diagonal) gene sequences. Calculated using the MEGA3 software. The clades are as follows: 1. Clade 1—Chilean Patagonia; 2. Clade 2—West Antarctic Peninsula; 3. Clade 3—Burdwood Bank; and 4. Clade 4—Falkland Islands. [file ECE3-14-e70522-s001.docx]

**Supplementary material**

**Table S1.** GenBank accession numbers for octocoral DNA sequences used in phylogenetic analysis.

|  |  | **GenBank Access** | | | |
| --- | --- | --- | --- | --- | --- |
| **Specie name** | **Sampling area** | **Cox1** | | **mtMutS** | **28S** |
| *Gersemia rubiformis^1^* | Northern Hemisphere | GQ342390 | | GQ342474 | JX203648 |
| *Gersemia juliepackardae^3^* | Northern Hemisphere | JX203820 | | JX203768 | JX203647 |
| *Gersemia antarctica^3,4^* | Antarctic | GQ342389 | | GQ342473 | JX203646 |
| *Alcyonium digitatum^3,4^* | North Atlantic and Mediterranean | GQ342381 | | AY607777 | JX203641 |
| *Alcyonium siderium^2,3^* | North Atlantic and Mediterranean | GU355957 | | GU355973 | KF728090 |
| *Alcyonium variabile^4,5,3^* | South Africa | GQ342385 | | KF728095 | JX203646 |
| *Alcyonium dolium^6^* | South Africa | MG053073 | | MG053055 | MG053011 |
| *Alcyonium palmatum^3^* | North Atlantic and Mediterranean | GQ342382 | | GQ342467 | JX203643 |
| *Alcyonium glomeratum^2,7^* | North Atlantic and Mediterranean | GU355947 | | AY607776 | KF728091 |
| *Alcyonium hibernicum^2,7^* | North Atlantic and Mediterranean | GU355949 | | AY607771 | KF728089 |
| *Alcyonium corralloides^3,6^* | North Atlantic and Mediterranean | MT281286 | | MG053055 | JX203640 |
| *Alcyonium bocagei^2,7^* | North Atlantic and Mediterranean | GU355943 | | GU355960 | KF728088 |
| *Alcyonium haddoni^2,3^* | South America | GU355958 | | GU355974 | JX203642 |
| *Parerythropodium grandiflorum^8^* | Northern Hemisphere | - | | KU712083 | - |
| *Alcyonium aurantiacum^9^* | New Zealand | - | | DQ302806 | - |
| *Ushanaia fervens^5^* | New Zealand | - | | OM891043 | - |
| *Ushanaia solida^5^* | New Zealand | - | | OM891046 | - |
| *Azoriella bayeri^1,3^* | Northern Hemisphere | GQ342407 | | GQ342486 | JX203672 |
| *Alcyonium sp^10^* | Falkland Islands | OP797670 | | PP182331 | OP799354 |
| *Alcyonium haddoni^10^* | Pia Fjord, Chile | OP797671 | | PP182332 | OP799355 |
| *Alcyonium haddoni^10^* | Barry Fjord, Chile | OP797672 | | PP182333 | OP799356 |
| *Alcyonium haddoni^10^* | San Isidro, Chile | OP797673 | | PP182334 | OP799357 |
| *Alcyonium haddoni^10^* | Punta Ganso, Chile | PP101875 | | PP182336 | OR785765 |
| *Alcyonium haddoni^10^* | Marta, Chile | PP101876 | | PP182337 | OR785766 |
| *Alcyonium haddoni^10^* | Metri, Chile | OP797674 | | PP182335 | OP799358 |
| *Alcyonium sp^10^* | Bordwood Bank | OP797669 | | PP182330 | OP799353 |
| *Alcyonium antarcticum^10^* | Rothera, Antarctic | OP797666 | | PP182327 | OP799350 |
| *Alcyonium antarcticum^10^* | Yelcho, Antarctic | OP797667 | | PP182328 | OP799351 |
| *Alcyonium antarcticum^10^* | Fildes Bay, Antarctic | | OP797668 | PP182329 | OP799352 |

Reference: (^1^Brockman & McFadden, 2012; ^2^McFadden *et al.*, 2011; ^3^McFadden & Van Ofwegen, 2012; ^4^Parrin *et al*, 2012, ^5^ Kessel. *et al.* , ^6^McFadden & Van Ofwegen, 2017, ^7^ McFadden & Van Ofwegen, 2013, ^8^Lawler *et al.*, 2016, ^9^McFadden et al, 2006 , ^10^This paper)

**Table S2.** Pairwise genetic distance matrix based on mtDNA (Cox1 + mtMutS; below the diagonal) and 28S rDNA (above the diagonal) gene sequences. Calculated using the MEGA3 software. The clades are as follows: 1. Clade 1 – Chilean Patagonia; 2.Clade 2 - West Antarctic Peninsula; 3.Clade 3 - Burdwood Bank; and 4. Clade 4 - Falkland Islands. This information is illustrated in Figures 3 and 4.

|  | **1** | **2** | **3** | **4** |
| --- | --- | --- | --- | --- |
| **1. Clade 1** | | 0.012 | 0.013 | 0.014 |
| **2. Clade 2** | 0.009 |  | 0.006 | 0.014 |
| **3. Clade 3** | 0.010 | 0.014 |  | 0.013 |
| **4. Clade 4** | 0.004 | 0.008 | 0.010 |  |
